# The use of progeroid DNA repair-deficient mice for assessing anti-aging compounds, illustrating the benefits of nicotinamide riboside

**DOI:** 10.3389/fragi.2022.1005322

**Published:** 2022-10-12

**Authors:** María B. Birkisdóttir, Ivar van Galen, Renata M. C. Brandt, Sander Barnhoorn, Nicole van Vliet, Claire van Dijk, Bhawani Nagarajah, Sandra Imholz, Conny T. van Oostrom, Erwin Reiling, Ákos Gyenis, Pier G. Mastroberardino, Dick Jaarsma, Harry van Steeg, Jan H. J. Hoeijmakers, Martijn E. T. Dollé, Wilbert P. Vermeij

**Affiliations:** ^1^ Princess Máxima Center for Pediatric Oncology, Utrecht, Netherlands; ^2^ Oncode Institute, Utrecht, Netherlands; ^3^ Department of Molecular Genetics, Erasmus MC Cancer Institute, Erasmus University Medical Center, Rotterdam, Netherlands; ^4^ Department of Hematology, Erasmus University Medical Center, Rotterdam, Netherlands; ^5^ Centre for Health Protection, National Institute for Public Health and the Environment, (RIVM), Bilthoven, Netherlands; ^6^ Faculty of Medicine, CECAD, Institute for Genome Stability in Aging and Disease, University of Cologne, Cologne, Germany; ^7^ IFOM-The FIRC Institute of Molecular Oncology, Milan, Italy; ^8^ Department of Life, Health, and Environmental Sciences, University of L'Aquila, L'Aquila, Italy; ^9^ Department of Neuroscience, Erasmus University Medical Center, Rotterdam, Netherlands

**Keywords:** aging, DNA damage repair, anti-aging interventions, pharmacological screening, dietary restriction mimetics, NAD, progeria

## Abstract

Despite efficient repair, DNA damage inevitably accumulates with time affecting proper cell function and viability, thereby driving systemic aging. Interventions that either prevent DNA damage or enhance DNA repair are thus likely to extend health- and lifespan across species. However, effective genome-protecting compounds are largely lacking. Here, we use *Ercc1* ^Δ/−^ and *Xpg* ^−/−^ DNA repair-deficient mutants as two *bona fide* accelerated aging mouse models to test propitious anti-aging pharmaceutical interventions. *Ercc1* ^Δ/−^ and *Xpg* ^−/−^ mice show shortened lifespan with accelerated aging across numerous organs and tissues. Previously, we demonstrated that a well-established anti-aging intervention, dietary restriction, reduced DNA damage, and dramatically improved healthspan, strongly extended lifespan, and delayed all aging pathology investigated. Here, we further utilize the short lifespan and early onset of signs of neurological degeneration in *Ercc1* ^Δ/−^ and *Xpg* ^−/−^ mice to test compounds that influence nutrient sensing (metformin, acarbose, resveratrol), inflammation (aspirin, ibuprofen), mitochondrial processes (idebenone, sodium nitrate, dichloroacetate), glucose homeostasis (trehalose, GlcNAc) and nicotinamide adenine dinucleotide (NAD^+^) metabolism. While some of the compounds have shown anti-aging features in WT animals, most of them failed to significantly alter lifespan or features of neurodegeneration of our mice. The two NAD^+^ precursors; nicotinamide riboside (NR) and nicotinic acid (NA), did however induce benefits, consistent with the role of NAD^+^ in facilitating DNA damage repair. Together, our results illustrate the applicability of short-lived repair mutants for systematic screening of anti-aging interventions capable of reducing DNA damage accumulation.

## Introduction

With the rise of the average age of the population and in parallel the prevalence of age-related disorders, such as cancer and neurodegeneration, the search for interventions or compounds counteracting aging and age-related processes has gained considerable interest. Whereas aging is considered a multifactorial process (López-Otín et al., 2013), accumulation of DNA damage, which is intrinsic to life, plays a major role (Hoeijmakers, 2009; Niedernhofer et al., 2018; Schumacher et al., 2021). Interventions that preserve the genome by preventing DNA damage or enhancing DNA repair are thus likely to result in extended health- and lifespan across species, but are still largely lacking (Partridge et al., 2020; Petr et al., 2020). This might be partly due to the fact that evaluation whether a compound extends health- and lifespan of wildtype (WT) mammals involves large numbers of animals and requires expensive, labor-intensive and lengthy experiments. Additionally, it is not always clear what aging process/es are targeted when a drug has a beneficial effect. These issues can, to a significant extent, be circumvented by using DNA repair-deficient, accelerated aging mouse mutants. *Ercc1* ^Δ/−^ and *Xpg* ^−/−^ mice are two well-characterized DNA repair mutants that exhibit widespread premature aging across many tissues within a lifespan of 4–6 months (Dollé et al., 2011; Barnhoorn et al., 2014). ERCC1 and XPG (also known as ERCC5) are endonucleases involved in different DNA repair pathways, that each resolve specific types of DNA lesions. Both cut the damaged DNA strand at opposing sites during the essential excision step in the global genome nucleotide excision repair (GG-NER) and transcription-coupled repair (TCR) processes, which removes the wide class of bulky, helix-distorting DNA lesions respectively genome-wide or after they arrest elongating RNA polymerase complexes, interfering with transcription (Marteijn et al., 2014; Vougioukalaki et al., 2022). Additionally, ERCC1 is implicated in interstrand crosslink repair and single-strand annealing (that repair DNA crosslinks and double strand DNA breaks respectively), while XPG is also involved in base excision repair (for the removal of subtle, oxidative lesions) (Klungland et al., 1999; Trego et al., 2016; Faridounnia et al., 2018; Apelt et al., 2021). As a result of their mutation, a broad variety of DNA lesions accumulate more rapidly in these mice, causing genomic instability, functional decline and premature aging. In particular post-mitotic tissues and more slowly dividing cells rely largely on TCR and thus accumulate transcription-blocking DNA lesions, which impede transcription elongation and cause transcription stress driving functional decline (Vermeij et al., 2016; Lans et al., 2019).

We recently showed that dietary restriction (DR, also known as caloric restriction; CR), largely extended health- and lifespan of progeroid *Ercc1* ^Δ/−^ and *Xpg* ^−/−^ DNA repair-deficient mice by alleviating DNA damage and transcription stress (Vermeij et al., 2016). Meanwhile, rapamycin, an inhibitor of the mTOR pathway and an alleged DR-mimetic compound that also has anti-aging effects in WT animals showed no benefits (Ferrara-Romeo et al., 2020; Birkisdóttir et al., 2021). This indicates that DR, but not rapamycin, works, at least partly, by relieving genotoxic/transcription stress (Vermeij et al., 2016; Birkisdóttir et al., 2021).

Here we assessed the effect of several compounds on the short lifespan and early onset neurological abnormalities in *Ercc1* ^Δ/−^ and *Xpg* ^−/−^ mice, illustrating them as useful models for the screening of (pharmaceutical) interventions that increase genome stability and prevent aging. We focus on a set of compounds that have shown to extend lifespan in WT mice, including several (resveratrol, metformin, acarbose, and aspirin) tested in the multi-institutional NIA Intervention Testing Program (Strong et al., 2008; Miller et al., 2011; Nadon et al., 2016; Strong et al., 2016). In parallel, we test various compounds, that have shown health benefits in different disease models, to investigate whether they provide protection in a DNA repair-deficient background. This includes drugs that adjust redox status, mitochondrial functioning and metabolism. Lastly, we test two nicotinamide adenine dinucleotide (NAD^+^) precursors; nicotinamide riboside (NR) and nicotinic acid (NA), that have shown broad anti-aging indications (Xie et al., 2020; Imai and Guarente, 2014; Scheibye-Knudsen et al., 2014; Abdellatif et al., 2021a). As DR resulted in more than doubling of the lifespan of progeroid DNA repair-deficient mice (Vermeij et al., 2016), we anticipated that compounds influencing genomic instability would yield large differences in lifespan in these mouse models and therefore used low sample sizes to screen for a larger set of compounds. This will further confirm whether compounds known to enhance DNA repair would, as to be expected, benefit DNA repair-deficient mouse models.

## Materials and methods

### Mouse models and interventions

The generation of *Ercc1* ^Δ/−^ and *Xpg* ^−/−^ mice (both in genetically uniform F1 C57BL6J/FVB hybrid background) has been previously described (Weeda et al., 1997; Barnhoorn et al., 2014). Shortly, *Ercc1* ^Δ/−^ mice were obtained by crossing Ercc1^Δ/+^ with Ercc1^+/−^mice (both in either in a pure FVB or C57BL6J background), yielding mice with one C-terminally truncated “delta” *Ercc1* allele (*Ercc1* ^Δ^) and one knock-out allele (*Ercc1* ^-^) (Weeda et al., 1997; Dollé et al., 2011; Vermeij et al., 2016). *Xpg* ^/−^ mice were generated similarly by crossing Xpg^+/−^ from a pure C57BL6J background with Xpg^+/−^ mice from a pure FVB background, yielding mice fully deficient in *Xpg* (Barnhoorn et al., 2014). Hence, all animals used in the studies described here were of the same F1 C57BL6J/FVB hybrid background. Typical unfavorable characteristics, such as blindness in an FVB background or deafness in a C57BL6J background, are ameliorated in this hybrid background. Animals were group-housed at both RIVM (Rijksinstituut voor Volksgezondheid en Milieu/National Institute for Public Health and the Environment) and EMC (Erasmus Medical Center University Rotterdam) locations, unless single housing was preferred for the specific intervention, and kept in ventilated cages under SPF conditions. Environmental conditions were controlled with temperature of 20–22°C and 12 h light:12 dark cycles. All animals were bred and maintained on AIN93G synthetic pellets (Research Diet Services B.V., Wijk bij Duurstede, Netherlands; gross energy content 4.9 kcal/g dry mass, digestible energy 3.97 kcal/g). Since the *Ercc1* ^Δ/−^ and *Xpg* ^−/−^ mice are smaller, food was administered within the cages and water bottles with long nozzles were used from around 2 weeks of age. Mice had free access to food and water. Mice were weighed weekly and visually inspected daily for moribund states (or serious illnesses exceeding the common aging trajectories of the mouse models used). Experiments were performed in accordance with the Principles of Laboratory Animal Care with guidelines approved by the Dutch Ethical Committee in full accordance with European legislation (DEC no. 200900263, 201100026, and 2016-0047-011 for RIVM and DEC no. 139-12-13 and 139-12-18 for EMC). To further facilitate a faster assessment of the potential anti-aging compounds in DNA repair-deficient animals we divided interventions over male and female *Ercc1* ^Δ/−^ and *Xpg* ^−/−^ mice and used in parallel two separate testing locations that previously gave similar experimental results (Vermeij et al., 2016; Birkisdóttir et al., 2021). Group sizes were limited in line with the 3R’s and based on previous reproducible results (Vermeij et al., 2016; Birkisdóttir et al., 2021).

### Pharmaceutical interventions

An overview of the experiments including the compounds and dosages used is shown in Supplementary Table S1. At the RIVM, compounds were supplemented in the food of *Ercc1* ^Δ/−^ mice to the concentrations indicated. Food intake and bodyweight were measured weekly. For experiments conducted at the EMC, drugs were dissolved in the drinking water of *Ercc1* ^Δ/−^ and *Xpg* ^−/−^ mice. In addition to food intake and bodyweight, water intake was measured every week in the EMC. The number of animals and gender per experiment is indicated in Supplementary Table S1 and in Figure 4.

### Phenotype scoring

All animals in experiments at the EMC were scored weekly for neurological abnormalities. Mice were scored positive for whole body tremors if they trembled for at least 10 s when put on a flat surface for a total of 20 s. During the same period, imbalance was measured. Mice that could not maintain an upright orientation during the 20 s period were scored as having imbalance.

### Statistical analysis

All statistical measurements were made with GraphPad Prism (version 8.0.2). One-Way-ANOVA, with Dunnet’s multiple comparison test was used to estimate differences in experimental groups vs. control groups for bodyweight, food, and water intake. Unpaired t-test was used to estimate difference between the control and idebenone group. Log-rank (Mantel-Cox) tests were used to compare curves for onset of tremors, imbalance and survival. Area under the curve (AUC) for bodyweight was calculated as the value of log10 between weeks 6 and 16, when at least 75% of all groups were still alive. Outliers of AUC bodyweight measures were identified and removed using the Grubb method, with alpha set at 0,01 (in total one animal in *Xpg* ^−/−^ control group). Two-Way-ANOVA with Sidák’s post hoc multiple comparisons test was used to assess effects of gender on treatment efficiency over the different mouse models and testing sites. Hazard plots show log values of hazard ratio (logrank), when compared to control group and 95% confidence interval. Graphs illustrate mean ± standard deviation.

## Results

### Nutrient sensing modulators

**FIGURE 1:**
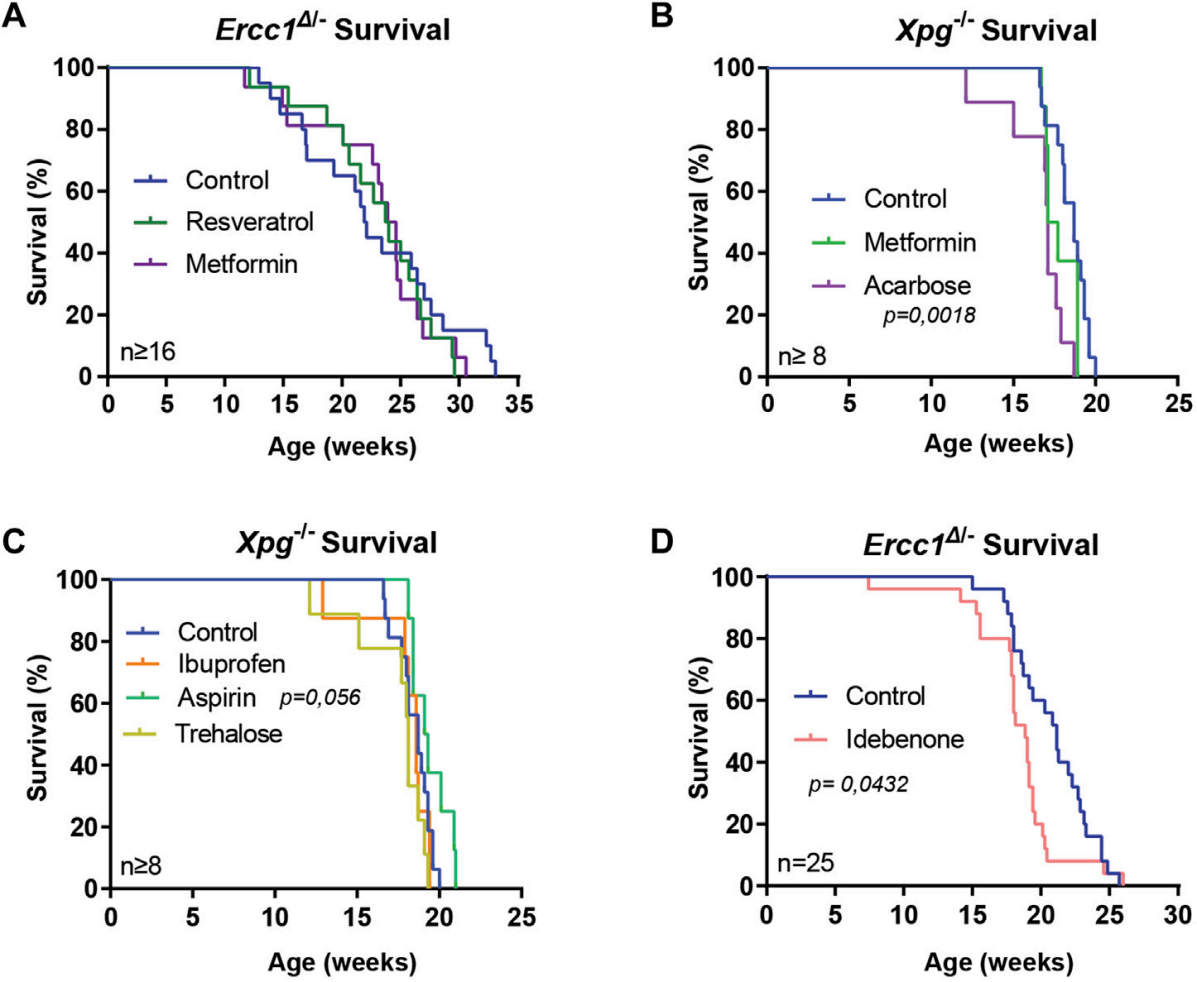
Supplementation of compounds for assessing lifespan changes of DNA repair-deficient mice. **(A–D)**, Survival curves of *Ercc1* ^Δ/−^ mice on resveratrol, metformin **(A)** or idebenone **(D)** supplemented diet and *Xpg* ^−/−^ mice treated with metformin, acarbose **(B)**, aspirin or ibuprofen **(C)**. Indicated values are Mean (±SD). Significant *p* values of log-rank survival test **(B,D)** are indicated.

Manipulation of a number of nutrient signaling pathways, including GH/IGF1, mTOR, and AMP-kinase (AMPK) has revealed that they control lifespan in many model organisms, including mice. This has triggered research on anti-aging features of small molecules that target these pathways, including rapamycin, resveratrol, and metformin (Howitz et al., 2003; Miller et al., 2011; Martin-Montalvo et al., 2013; Strong et al., 2013; Kennedy and Pennypacker, 2014; Strong et al., 2016; Gonzalez-Freire et al., 2020; Wilson et al., 2021). Recently we found that the mTOR inhibitor rapamycin, that extends medium and maximum lifespan in WT mice, for yet unclarified reasons, failed to do so in *Ercc1* ^Δ/−^ and *Xpg* ^−/−^ mice (Miller et al., 2011; Birkisdóttir et al., 2021). Here, we tested metformin that extends lifespan in C57/Bl6 mice but not in UM-HET3 WT mice and whose anti-aging effects have been primarily linked to the activation of AMPK signaling (Hardie et al., 2012; Martin-Montalvo et al., 2013; Strong et al., 2016). Using two genetic backgrounds and two manners of administration we supplemented metformin to the food of *Ercc1* ^Δ/−^ mice and to the water of *Xpg* ^−/−^ mice. At the same time acarbose, an alpha-glucosidase inhibitor that lowers postprandial glucose levels and extends lifespan in UM-HET3 mice (Harrison et al., 2014; Strong et al., 2016; Harrison et al., 2019) and resveratrol, an activator of sirtuins, were tested in *Xpg* ^−/−^ and *Ercc1* ^Δ/−^ mice respectively. Food consumption and bodyweights were carefully monitored since these mutants are very sensitive to changes in caloric intake (Vermeij et al., 2016). Whereas these values did not differ for metformin and resveratrol groups, interestingly the body weight was higher in mice subjected to acarbose (Supplementary Figures S1, S2A–C). Water intake for *Xpg* ^−/−^ mice that received the compounds in water, was the same for all groups, and the onset of neurological abnormalities of tremors and imbalance was unaltered (Supplementary Figures S2D–F). Metformin and resveratrol did not influence lifespan of DNA repair-deficient mice, whereas *Xpg* ^−/−^ animals given acarbose surprisingly lived significantly shorter than control-fed animals (Figures 1A,B; *p* = 0,0018).

### Anti-inflammatory drugs

Nonsteroidal anti-inflammatory drug (NSAID), such as aspirin and ibuprofen that operate by inhibiting prostaglandin synthesis *via* inhibition of cyclooxygenases (Smith and Marnett, 1991), have shown to have the ability to extend health- and lifespan in a variety of organisms (Bulckaen et al., 2008; Strong et al., 2008; Wan et al., 2013; He et al., 2014; Saleh et al., 2014; Song et al., 2017). We tested both drugs in *Xpg* ^−/−^ mice, by supplementing the compounds to drinking water. Neither ibuprofen nor aspirin influenced water intake, food intake or bodyweight, and onset of tremors and imbalance remained unchanged (Supplementary Figure S3). Only aspirin supplementation showed a trend towards increased medium and maximum lifespan (Figure 1C; *p* = 0.056).

### Anti-oxidants

Oxidative stress resulting in DNA damage has been repeatedly linked to aging, but anti-oxidant interventions have shown limited success (Møller and Loft, 2004; Gutteridge and Halliwell, 2010; Sadowska-Bartosz and Bartosz, 2014). To test directly the effect of anti-oxidants in a mouse model of accelerated DNA damage accumulation we supplemented idebenone, a synthetic mild anti-oxidant of the coenzyme Q family to the food of *Ercc1* ^Δ/−^ mice (Geromel et al., 2002). Interestingly, food intake of mice receiving idebenone was slightly elevated compared to control-fed animals (Supplementary Figure S4A; *p* = 0.004) but without altering bodyweight (Supplementary Figures S4B,C). In addition, lifespan of treated mice was slightly shortened (Figure 1D; *p* = 0.0432). These results align with earlier studies using anti-oxidant treatment in *Ercc1* ^Δ/−^ (Milanese et al., 2019), indicating that benefits of externally provided anti-oxidants are, just as for WT organisms, often limited or even detrimental.

### Mitochondrial and glucose homeostasis modifiers

**FIGURE 2:**
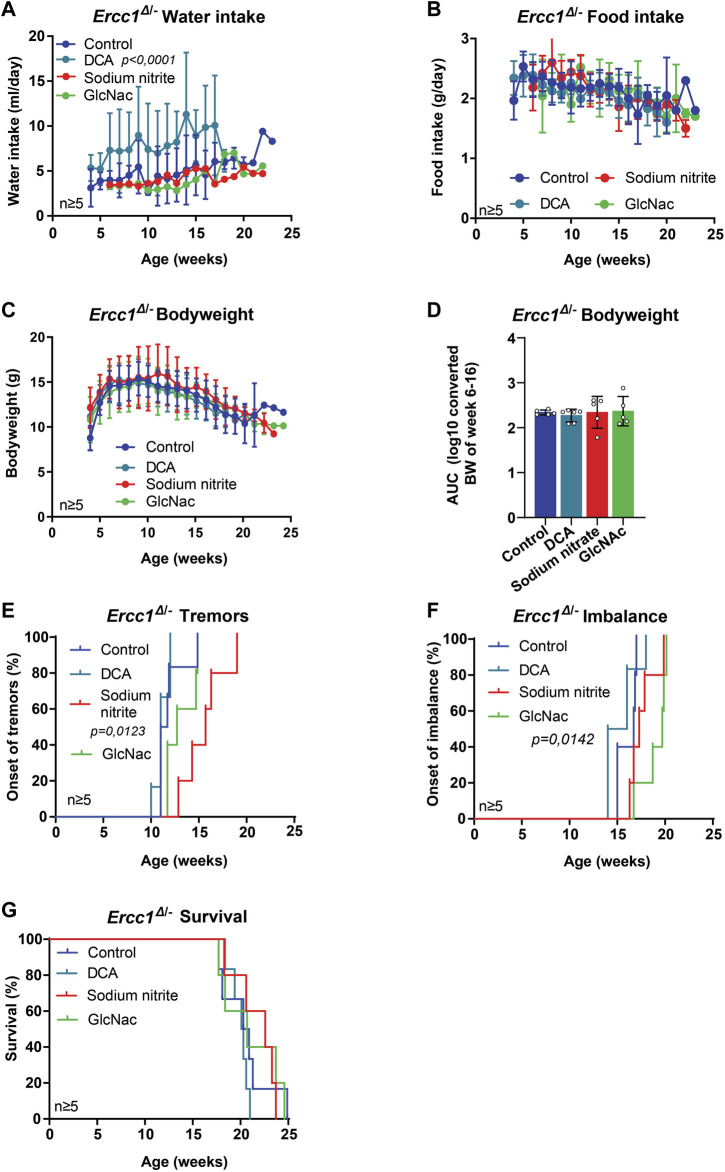
The effect of mitochondrial modifiers and GlcNAc supplementation on the lifespan and onset of neurological phenotypes of *Ercc1* ^Δ/−^ mice. **(A–D)**, Comparison between *Ercc1* ^Δ/−^ control mice and animals supplemented with sodium nitrite, DCA, or GlcNAc on water intake **(A)**, food intake **(B)**, bodyweight over time **(C)** and body weight as area under the curve (AUC) of measurements between 6–16 weeks **(D)**. **(E–G)**, Onset of neurological abnormalities; tremors **(E)** and imbalance **(F)**, and survival **(G)** of the same animals. Indicated values are Mean (±SD). Significant *p* values of the Dunnet’s multiple comparison test **(A,E,F)** are indicated.

Next, we used our mouse models to test several drugs that have shown some health benefits in different disorder models, but have not been intensively studied in mammals for lifespan extension. This included sodium dichloroacetate (DCA), a compound that improves the functional status of mitochondria through the stimulation of the pyruvate dehydrogenase (PDH) activity, and has shown health benefits in animals, including in nematodes and in an Amyotrophic Lateral Sclerosis (ALS) mouse model (Schaffer et al., 2011; Miquel et al., 2012). Furthermore, we tested sodium nitrite that might improve mitochondrial function by reversible S-nitrosation of complex one and has shown to provide protective effects during hypoxic conditions of ischemia/reperfusion and for models of Parkinson’s disease (Higashiyama, 2002; Raat et al., 2009; Lundberg et al., 2015; Milanese et al., 2018). *Ercc1* ^Δ/−^ mice treated with DCA showed high variation but in general increased water intake (Figure 2A; *p* < 0.0001), without influencing food intake, bodyweight nor the onset of neurological phenotypes or survival (Figures 2B–G). Mice treated with sodium nitrite did not differ in water or food intake or in bodyweight (Figures 2A–D). Whereas there was no effect on imbalance or lifespan (Figures 2F,G) the mice did show a significant delay in the onset of tremors (Figure 2E; *p* = 0.0123).

Additionally, two groups received different kinds of sugars, both known to alter post-translational modifications of proteins, with GlcNAc being supplemented to *Ercc1* ^Δ/−^ and trehalose to *Xpg* ^−/−^ mice. GlcNAc is an amide derivative of glucose, and trehalose a disaccharide (Kamel et al., 1991; Wani et al., 2017). Expression of O-GlcNAcylated proteins change with age, especially in the brain, and might thus provide a target for treatment of neurodegenerative disorders (Denzel et al., 2014; Wani et al., 2017), whereas trehalose has been shown to stabilize protein conformation, suppress aggregation of unfolded proteins and reduce polyglutamine aggregates in Huntington mice (Singer and Lindquist, 1998; Tanaka et al., 2004; Khalifeh et al., 2021). Neither intervention changed water intake, food intake, bodyweight, tremors nor lifespan (Figures 1C, 2A–E,G, Supplementary Figures S5A–E). Trehalose had, in addition, no effect on imbalance (Supplementary Figure S5F), whereas GlcNAc appeared to delay its onset significantly compared to other groups (Figure 2F; *p* = 0.0142)

### Nicotinamide adenine dinucleotide (NAD^+^) precursors

**FIGURE 3:**
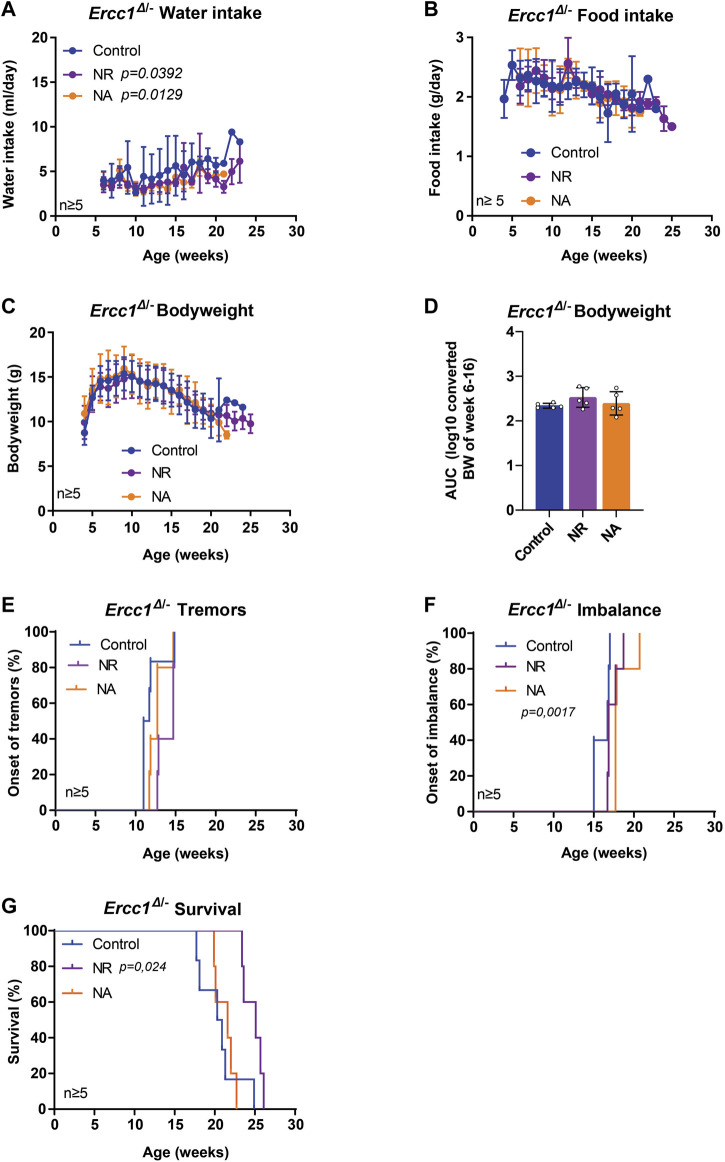
The effect of nicotinamide adenine dinucleotide (NAD^+^) precursor ribosome supplementation on the lifespan and onset of neurological phenotypes of *Ercc1* ^Δ/−^ mice. **(A–C)**, Values of water intake **(A)**, food intake **(B)** body weight changes over time **(C)** and body weight as area under the curve (AUC) **(D)** in control animals and animals receiving supplementation of two NAD^+^ precursors; NA and NR. **(E–F)**, Onset of neurological abnormalities; tremors **(E)** and imbalance **(F)** with age of *Ercc1* ^Δ/−^ control mice and mice receiving NR or NA. **(G)**. Survival curve showing lifespan of the three groups. Indicated values are Mean (±SD). Significant *p* values of the Dunnet’s multiple comparison test **(A)** and log-rank survival test **(F,G)** are indicated.

NAD^+^ and NAD^+^ precursors represent a group of compounds with increasing reputation in anti-aging research (Gonzalez-Freire et al., 2020). NAD^+^ is an essential metabolite central to many cellular processes, including the regulation of DNA repair. NAD^+^ levels are known to decline with age with numerous studies showing that supplementation with NAD^+^ precursors can improve health parameters across tissues and even extend lifespan (Yoshino et al., 2018; Xie et al., 2020; Abdellatif et al., 2021b). Here, we assessed two key NAD^+^ precursors, nicotinic acid (NA) and nicotinamide riboside (NR), each enhancing NAD^+^ biosynthesis through different routes, i.e. *via* the NAMN/NAAD or the NMN route respectively (Okabe et al., 2019). The mean intake of water was slightly lower in treated animals, but highly variable, with no difference from controls in food and bodyweight (Figures 3A–D). Interestingly, mice supplemented with NA revealed a significant delay in the onset of imbalance (Figure 3F, *p* = 0.0017), whereas NR supplementation showed a trend towards delaying the median age of onset of tremors (Figure 3E). Furthermore, NR significantly extended median lifespan by approximately 25% or 5 weeks, while animals receiving NA lived about as long as control-fed animals (Figure 3G; *p* = 0.024).

**FIGURE 4:**
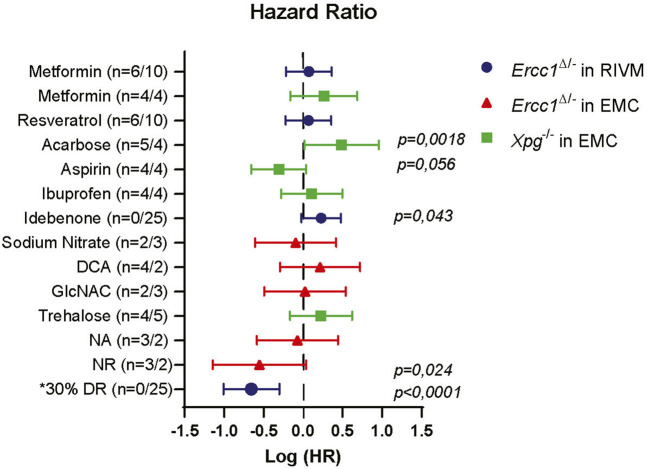
Comparison of lifespan changes induced in NER deficient mice. Forest plot showing the effectiveness of different intervention on extending lifespan in *Ercc1* ^Δ/−^ and *Xpg* ^−/−^ mice across two testing location. Plotted is the logarithm of hazard ratio (HR) with 95% logarithm confidence intervals as error bars when each treatment group is compared to its relevant control group. Lifespan extension was noted upon supplementation with NR. Significant (and close to) logrank (Mantel-Cox) *p* values are shown. Log(HR) below 0 indicate extension in lifespan and above 0 decline in lifespan. Number in brackets indicate number of animals (male/female). *Hazard ratio of DR group is calculated from Birkisdóttir et al. (2021).

Together, these data support the use of progeroid DNA repair-deficient mice as tools for assessing anti-aging compounds on lifespan or long-term health effects and for obtaining a better understanding of interventions potentially interfering with genomic instability. More importantly, we identified compounds, in addition to DR, able to extend life- and health span of progeroid DNA repair-deficient mice (Figure 4), potentially by affecting genomic instability.

## Discussion

To date, few interventions are known to delay or reduce DNA damage accumulation, a major driver of aging (Partridge et al., 2020; Petr et al., 2020). Here, we propose the use of DNA repair-deficient mouse models as a screening tool for potential anti-aging compounds as these mice: 1) are short-lived and age highly homogeneously; 2) represent ideal models to study the link between DNA damage and aging; 3) show multimorbidity, i.e. many aging features/hallmarks of natural aging across tissues; and 4) respond extremely well to the most robust and universal anti-aging intervention: DR. Thus, only limited numbers of animals are needed to provide initial information on the effectiveness of an intervention in a relatively short time.

Utilizing these advantages we tested pilot-wise a large number of compounds with potential anti-aging and cytoprotective effect. Across the different mouse models and testing sites, we did not observe major effects of gender on treatment efficiency, except for the control group at the RIVM location (Supplementary Figure S6). Overall results indicate, that, just as in wild type animals, no intervention with a single compound comes close to the extension of health- and lifespan achieved by DR (Figure 4).

Metformin, acarbose, and to a lesser extend resveratrol have been indicated to extend health- and lifespan, the first two even in mice, although these compounds did not yield uniformly lifespan-extending results (Howitz et al., 2003; Miller et al., 2011; Martin-Montalvo et al., 2013; Strong et al., 2013; Harrison et al., 2014; Strong et al., 2016; Harrison et al., 2019; Partridge et al., 2020). In our repair mutants none had any significant beneficial effect on health or lifespan, with acarbose even shortening lifespan. The shortened lifespan might be explained by their increase in bodyweight as generally DR reduces bodyweight and increases lifespan. These results, together with our former study that showed no effect of rapamycin on health and lifespan, indicates that the manipulation of a single nutrient sensing pathway seems not sufficient to replicate the lifespan promoting effect seen by DR. Alternatively, our mouse mutants already display a DR-like response by themselves, without DR. It is possible that this part of the DR-induced survival response is recapitulated by (some of) these agents, resulting in lack of effectiveness (Schumacher et al., 2008).

In parallel, we studied the effect of two nonsteroidal anti-inflammatory drugs, but both *Ercc1* ^Δ/−^ and *Xpg* ^−/−^ mice show systemic inflammation (Dollé et al., 2011; Barnhoorn et al., 2014; Yousefzadeh et al., 2021). Whereas ibuprofen did not reveal any benefits, the supplementation of aspirin resulted in a slight, albeit non-significant lifespan extension. Importantly, aspirin also extends lifespan specifically in male heterozygous UT-HET3 mice and might even enhance DNA damage repair (Mammone et al., 2006; Strong et al., 2008). The contribution of aspirin for health and lifespan, however, requires further investigation.

We furthermore utilized our mouse models to screen for the possible genomic stability effects of several other compounds. We broadly categorize these drugs into anti-oxidants, mitochondrial modifiers and glucose metabolites. These compounds have been implicated to have some benefits in different disorder models, without clear health and lifespan extension in WT animals. As reported in an earlier study (Milanese et al., 2019), we here confirm that the supplementation of anti-oxidants of *Ercc1* ^Δ/−^ mice does not extend their lifespan. Furthermore, studies showing the benefits of anti-oxidants supplementation in WT animals are limited, which implies the importance of endogenous redox balance, which is boosted by DR, and questions the use of anti-oxidant supplementation to decrease oxidative stress and DNA damage (Møller and Loft, 2004; Gutteridge and Halliwell, 2010). Moreover, two mitochondrial modifiers; sodium dichloroacetate (DCA) and sodium nitrite, and two glucose metabolites; GlcNAc and trehalose, that are thought to alter post-translational protein modifications had no lifespan extending effect on progeroid mice. However, sodium nitrite delayed the onset of premature tremors by 5 weeks, that normally appear at around 10–11 weeks in *Ercc1* ^Δ/−^ mice, whereas GlcNAc delayed the onset of imbalance by 3 weeks, that the mice develop at around 15–16 weeks of age.

The specific protection of neurological abnormalities is of interest. First, it suggests that distinct processes drive the onset of those different abnormalities and second, it indicates that some compounds have a selective benefit in the face of DNA repair deficiency. Thus, they might particularly affect, e.g., postmitotic neurons, dorsal root ganglion, skeleton muscles [all likely to be implicated in tremors and imbalance (Alyodawi et al., 2019)], the central nervous system as a whole or other specific processes driving these abnormalities. However, before conclusions can be drawn, the experiments need to be replicated with larger sample sizes and include further histopathological, behavioral, physiological and molecular analysis.

Numerous hallmarks of aging are displayed in the progeroid mouse models, and whereas interventions targeting specific processes, e.g., senolytic drugs or stem cell transplantation have shown some tissue-specific benefits in the mice, none have successfully extended lifespan (Lavasani et al., 2012; Baar et al., 2017; Fuhrmann-Stroissnigg et al., 2017; Kim et al., 2020). Together with our current research, we can speculate that reducing only specific hallmarks of aging, which in the case of our mouse models is driven by insufficient DNA repair, is not enough to extend lifespan. Moreover, as the hallmarks of aging are intrinsically interconnected, changing one of the parameters might interrupt a sensitive balance displayed by another and even prove to be detrimental. This might be especially important for DNA repair-deficient animals which likely must modulate their cellular processes to optimize their chances of survival in lights of continuous endogenous transcriptional stress (Niedernhofer et al., 2006; Milanese et al., 2019).

Instead, compounds and interventions that have the capacity to reduce DNA damage accumulation at its core are the ones that will be identified using DNA repair-deficient models. This is likely reflected in the positive effects noted by two different NAD^+^ precursors, NA and NR. NAD^+^ has shown to decline with age and its supplementation has shown promising results both in normal and accelerated aging and in (segmental) aging disorders of post-mitotic tissues (Xie et al., 2020; Imai and Guarente, 2014; Scheibye-Knudsen et al., 2014; Abdellatif et al., 2021a; Maynard et al., 2015). NAD^+^ is central for cellular processes such as metabolism and mitochondrial function, but also acts as a substrate for cAPD-ribose synthases (CD38), sirtuin deacetylases and poly-ADP-ribose polymerases (PARPs), a DNA damage sensors which is critical to various DNA repair processes and a trigger for apoptosis (Xie et al., 2020). Thus, it remains a possibility, that even though the mice are genetically deficient in TCR that NAD^+^ supplementation boosts complimentary alternative PARP-mediated DNA repair processes or influences signals triggered by DNA damage.

NA and NR follow different pathways of being synthesized into NAD, and their oral bioavailability is likely to differ (Trammell et al., 2016; Mehmel et al., 2020). Furthermore, the subcellular uptake of the distinct NAD^+^ precursors across tissues might differ with flux analysis showing, e.g., that NA is unlikely to be used in the brain but rather in spleen, intestine, pancreas and liver (Liu et al., 2018; Xie et al., 2020; Hikosaka et al., 2021). This could explain the difference we noted in our results; whereas NA specifically delayed the onset of imbalance, NR significantly extended lifespan, beyond the maximum age reached by any of the other drugs tested (Figure 4).

Together, our results show that progeroid mouse models can be used as efficient, economical, and relatively rapid screening platforms for validation of drugs or interventions that target DNA damage accumulation and/or benefit insufficient DNA repair. Further studies of promising compounds with different dosages and increased numbers of animals are required to conclude drug effectiveness with more confidence. In this study, however, we put more emphasis on the screening of multiple compounds rather than a large sample size for each testing group, which still revealed benefits using only a limited number of animals. Additional interventions might target different aspects of nutritional signaling in hopes of replicating the effect seen during DR. This could include adjusting the composition of macronutrients, micronutrients and individual amino acids. In addition, combination therapies should be more commonly explored were animals are supplemented with compounds that act on different pathways (e.g., nutrient signaling and NAD^+^ biosynthesis), as initial indications provided beneficial effects both in mice and in other animals (Strong et al., 2016; Castillo-Quan et al., 2019). Other routes could be to induce hormesis or using genetic therapies to trigger better cellular maintenance or DNA repair. Further studies for supplementation of different NAD^+^ precursors and their beneficial effects considering insufficient DNA repair would moreover be warranted, especially for identifying mechanisms on how it might benefit different symptoms and human disorders and which types of DNA damage it affects. Of special importance, targeting NAD^+^ levels might prove to be a feasible way to prevent or slow down disease progression of patients with deficiencies in DNA repair genes causing syndromes of premature segmental aging.

Both DR and NAD^+^ are suggested to impact DNA damage and/or repair and result in lifespan extension in our DNA repair-deficient mice, thus confirming the mice as valuable model for screening (pharmaceutical) interventions that contribute to sustained genome stability either *via* decreased DNA damage production or by the promotion of DNA repair.

## Data Availability

The original contributions presented in the study are included in the article/Supplementary Material, further inquiries can be directed to the corresponding authors.

## Supplementary material

Supplementary Figure S1 Food intake and body weight *Ercc1* ^Δ/−^ mice is not changed upon resveratrol and metformin treatment. **(A,B)**, Mean (±SD) food intake **(A)** and body weight over time **(B)** of mice on control chow or with chow supplemented with resveratrol or metformin. **(C)**, Body weight of 6–16 weeks as area under the curve (AUC) for each condition.

Supplementary Figure S2 Acarbose and metformin supplementation does not extend neurological health and lifespan in *Xpg* ^−/−^ mice. **(A–C)**, Mean (±SD) food intake **(A)**, body weight over time **(B)** or calculated between 6–16 weeks as area under the curve (AUC) **(C)** of control mice and mice on acarbose and metformin treatment. **(D)** Water intake of all animals **(E,F)**, Onset of neurological tremors **(E)** and imbalance **(F)** in the three groups of animals. Significant *p* values of the Dunnet’s multiple comparison test **(C)** are indicated.

Supplementary Figure S3 Anti-Inflammatory drugs do not affect onset of tremors and imbalance in *Xpg* ^−/−^ mice. **(A–F)**, Comparison of control animals and animals receiving ibuprofen or aspirin regarding food intake **(A)**, body weight **(B)**, area under the curve (AUC) calculation of bodyweight **(C)**, water intake **(D)**, onset of tremors **(E)** and imbalance **(F)**.

Supplementary Figure S4 Mild anti-oxidant, idebenone, increases food intake without altering body weight in *Ercc1* ^Δ/−^ mice. **(A–C)**, Food intake **(A)**, body weight. development **(B)** and area under the curve (AUC) bodyweight of mice fed with control chow and mice fed with idebenone supplemented chow. Significant *p* values of unpaired t test **(A)** are indicated.

Supplementary Figure S5 Trehalose does not benefit neurological health in *Xpg* ^−/−^ mice. **(A–D)**, food intake **(A)** body weight over time **(B)**, body weight as calculated as area under the curve (AUC) **(C)** and water intake **(D)** of *Xpg* ^−/−^ mice with or without Trehalose supplementation. **(E, F)**, Survival curves showing time of onset of tremors **(E)** and imbalance **(F)** of the same animals.

Supplementary Figure S6 Statistical testing on the effect of gender on treatment efficiency per mouse model and testing location. **(A–C)**, two-way ANOVA for *Xpg* ^−/−^ mice at the EMC {**(A)**; [F (1, 45) = 1,016, p = 0.3187]}, *Ercc1* ^Δ/−^ mice at the RIVM {**(B)**; [F (1, 46) = 10,51, p = 0.0022]} and EMC {**(C)**; [F (1, 20) = 3,151, p = 0.0911]}. Significance of individual comparisons using Sidák post hoc test between genders of a treatment are indicated with only the controls *Ercc1* ^Δ/−^ mice at the RIVM being statistically significant (*p* = 0.0045). **(D, E)**, Forest plot showing the effectiveness of different intervention on extending lifespan in *Ercc1* ^Δ/−^ and *Xpg* ^−/−^ mice across two testing location separated in males **(D)** and females **(E)**. Plotted is the logarithm of hazard ratio (HR) with 95% logarithm confidence intervals as error bars when each treatment group is compared to its relevant control group.

Supplementary Table S1 Overview of compounds, experimental location, delivery method, intervention start, concentration of drugs and number of male/female animals of each experimental group.
