# Corrigendum: The use of progeroid DNA repair-deficient mice for assessing anti-aging compounds, illustrating the benefits of nicotinamide riboside

**DOI:** 10.3389/fragi.2022.1086552

**Published:** 2022-11-23

**Authors:** María B. Birkisdóttir, Ivar van Galen, Renata M. C. Brandt, Sander Barnhoorn, Nicole van Vliet, Claire van Dijk, Bhawani Nagarajah, Sandra Imholz, Conny T. van Oostrom, Erwin Reiling, Ákos Gyenis, Pier G. Mastroberardino, Dick Jaarsma, Harry van Steeg, Jan H. J. Hoeijmakers, Martijn E. T. Dollé, Wilbert P. Vermeij

**Affiliations:** ^1^ Princess Máxima Center for Pediatric Oncology, Utrecht, Netherlands; ^2^ Oncode Institute, Utrecht, Netherlands; ^3^ Department of Molecular Genetics, Erasmus MC Cancer Institute, Erasmus University Medical Center, Rotterdam, Netherlands; ^4^ Department of Hematology, Erasmus University Medical Center, Rotterdam, Netherlands; ^5^ Centre for Health Protection, National Institute for Public Health and the Environment, (RIVM), Bilthoven, Netherlands; ^6^ Faculty of Medicine, CECAD, Institute for Genome Stability in Aging and Disease, University of Cologne, Cologne, Germany; ^7^ IFOM-The FIRC Institute of Molecular Oncology, Milan, Italy; ^8^ Department of Life, Health, and Environmental Sciences, University of L'Aquila, L'Aquila, Italy; ^9^ Department of Neuroscience, Erasmus University Medical Center, Rotterdam, Netherlands

**Keywords:** aging, DNA damage repair, anti-aging interventions, pharmacological screening, dietary restriction mimetics, NAD, progeria

In the published article, there was an error in Figures 2, 3 as published. The panels in **Figure 2** are labelled **A, B, C, D, E, E, and F** and in **Figure 3 A, B, C, D, D, E, and F**, while both were initially intended as **A, B, C, D, E, F, G**.

**FIGURE 2:**
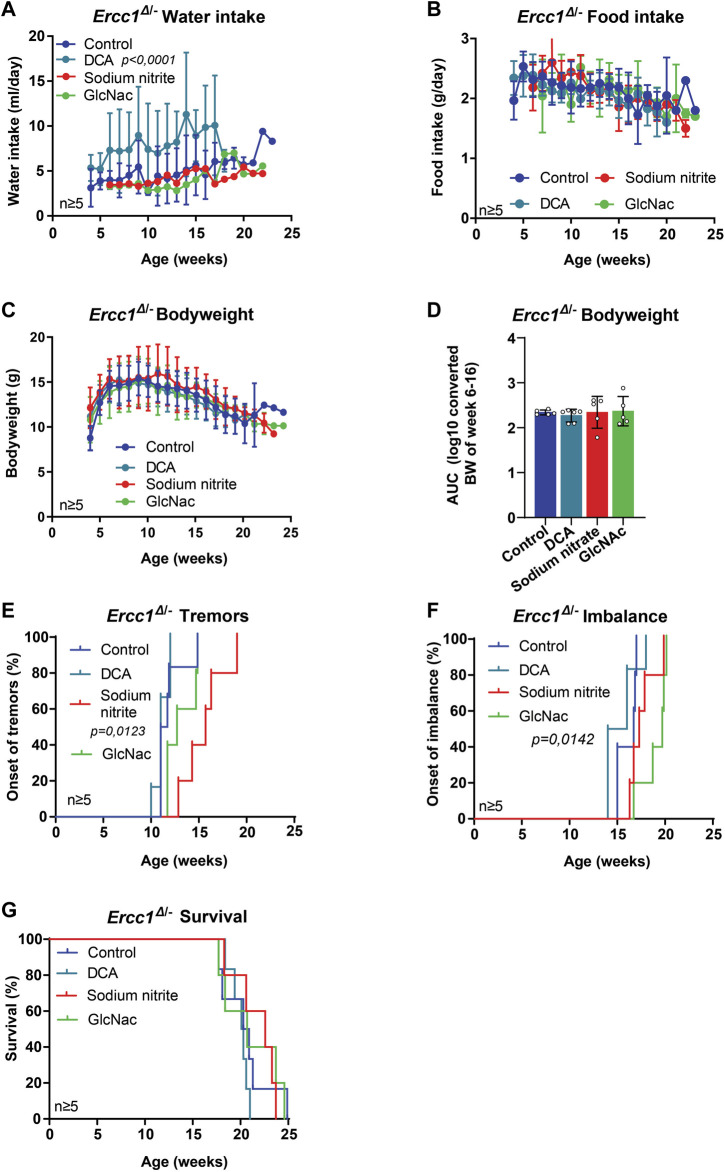
The effect of mitochondrial modifiers and GlcNAc supplementation on the lifespan and onset of neurological phenotypes of *Ercc1* ^Δ/−^ mice. **(A–D)**, Comparison between *Ercc1* ^Δ/−^ control mice and animals supplemented with sodium nitrite, DCA, or GlcNAc on water intake **(A)**, food intake **(B)**, bodyweight over time **(C)** and body weight as area under the curve (AUC) of measurements between 6–16 weeks **(D)**. **(E–G)**, Onset of neurological abnormalities; tremors **(E)** and imbalance **(F)**, and survival **(G)** of the same animals. Indicated values are Mean (±SD). Significant *p* values of the Dunnet’s multiple comparison test **(A,E,F)** are indicated.

**FIGURE 3:**
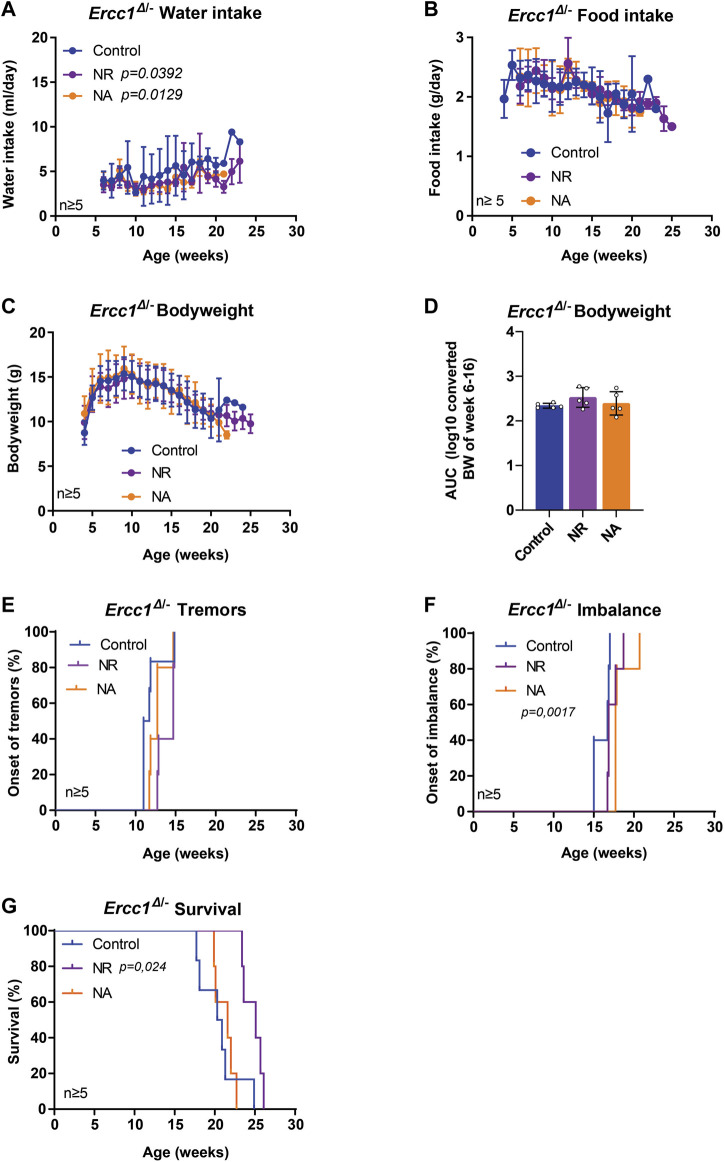
The effect of nicotinamide ribosome supplementation on the lifespan and onset of neurological phenotypes of *Ercc1* ^Δ/−^ mice. **(A–C)**, Values of water intake **(A)**, food intake **(B)** body weight changes over time **(C)** and body weight as area under the curve (AUC) **(D)** in control animals and animals receiving supplementation of two NAD^+^ precursors; NA and NR. **(E–F)**, Onset of neurological abnormalities; tremors **(E)** and imbalance **(F)** with age of *Ercc1* ^Δ/−^ control mice and mice receiving NR or NA. **(G)**. Survival curve showing lifespan of the three groups. Indicated values are Mean (±SD). Significant *p* values of the Dunnet’s multiple comparison test **(A)** and log-rank survival test **(F,G)** are indicated.

The corrected Figures 2, 3 appear below.

The authors apologize for this error and state that this does not change the scientific conclusions of the article in any way. The original article has been updated.

