# Supplementary figures and images for: The use of progeroid DNA repair-deficient mice for assessing anti-aging compounds, illustrating the benefits of nicotinamide riboside
Source: Front Aging. 2022 Oct 12;3:1005322. doi: 10.3389/fragi.2022.1005322 (PMC9596940; doi:10.3389/fragi.2022.1005322)

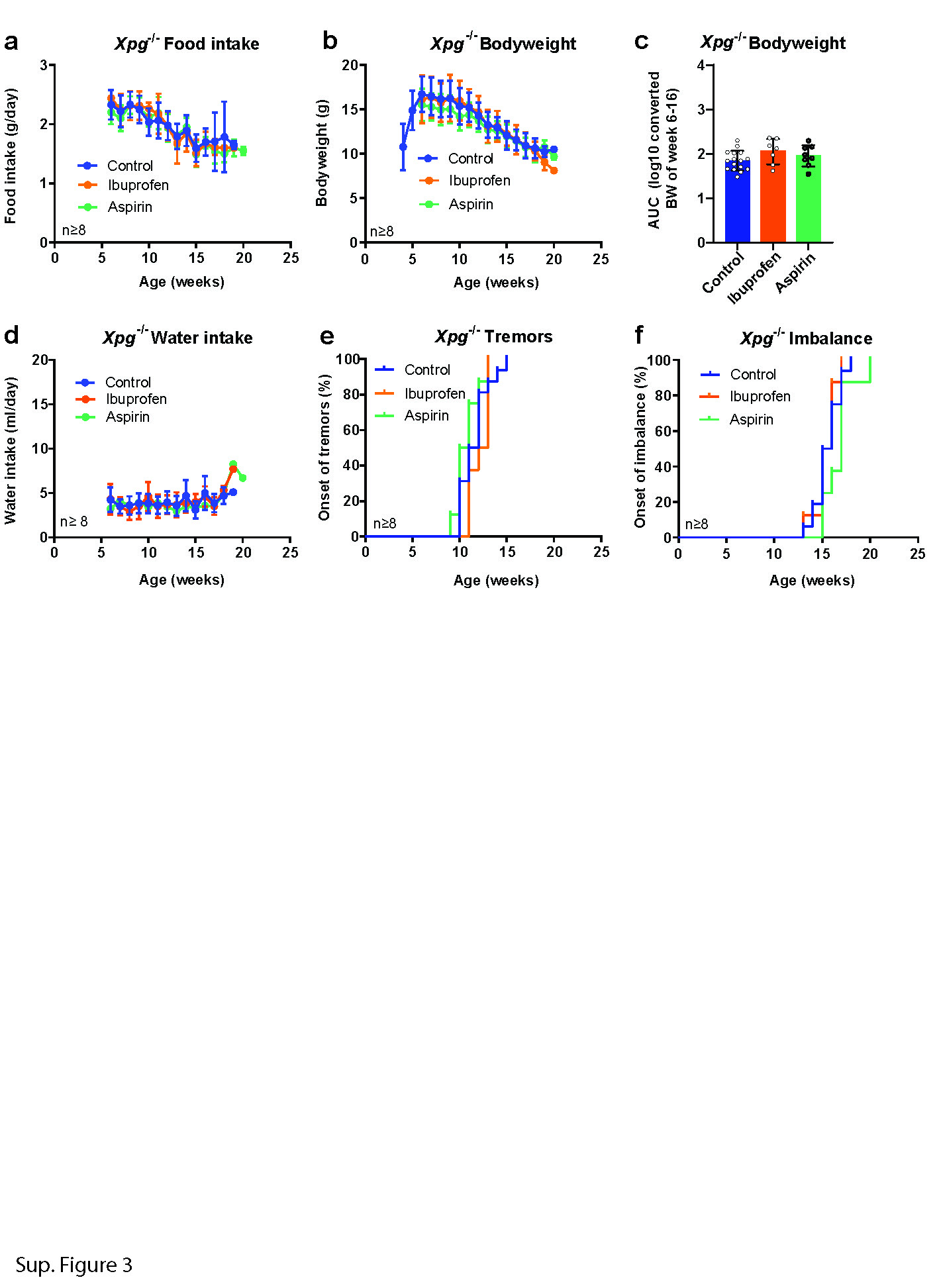

Supplement: Supplementary file 1 [file Image3.JPEG]

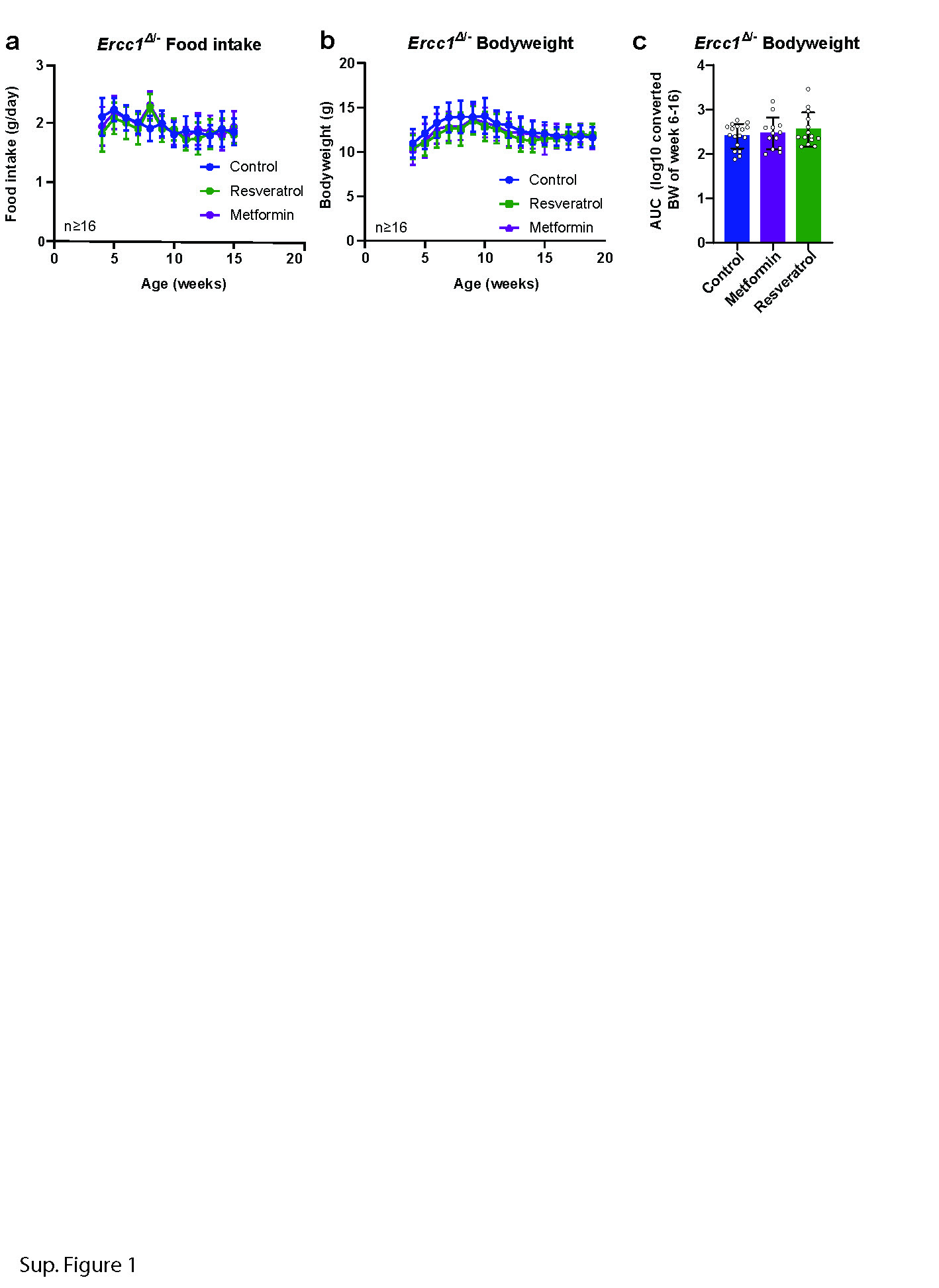

Supplement: Supplementary file 3 [file Image1.JPEG]

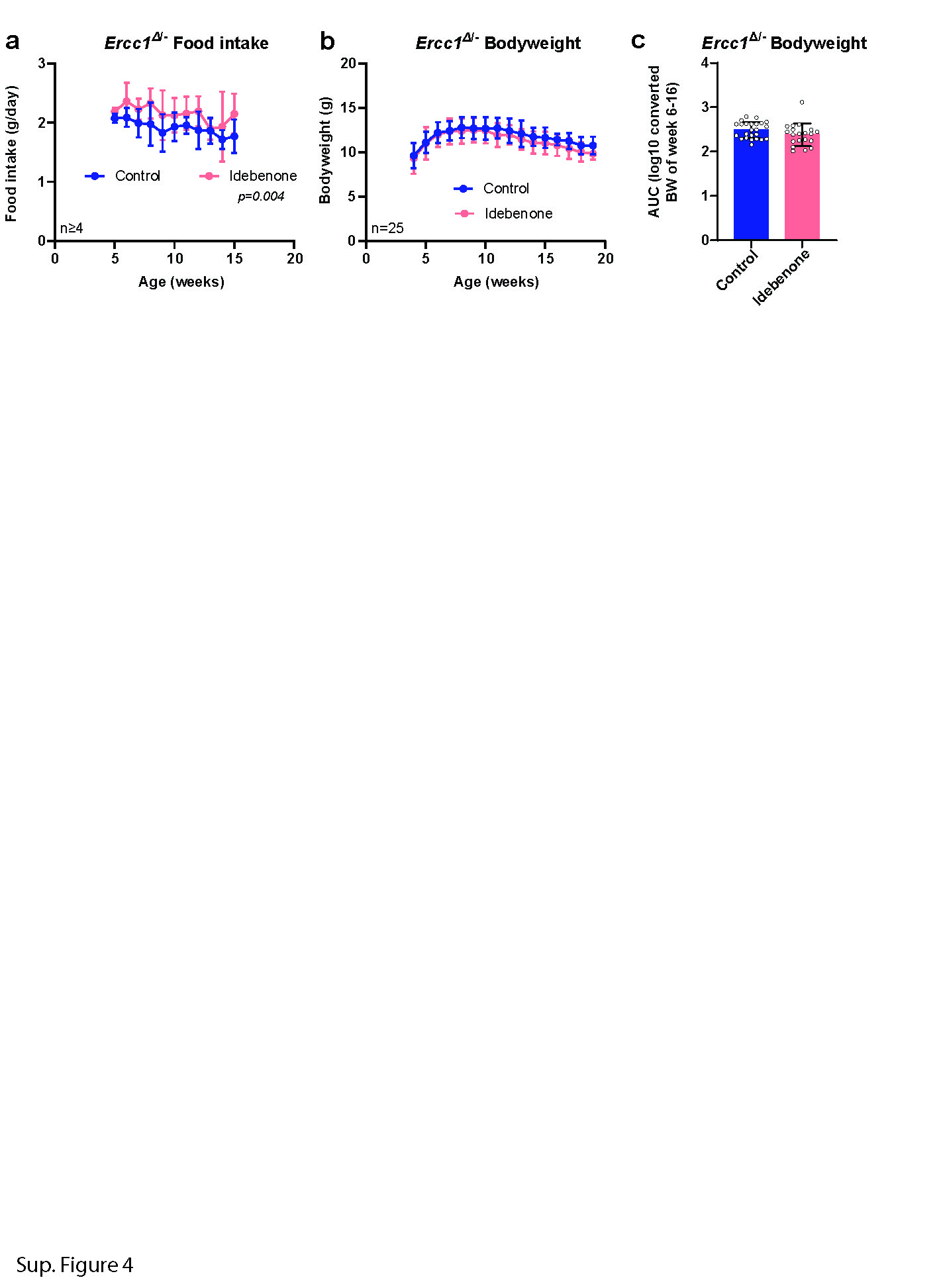

Supplement: Supplementary file 4 [file Image4.JPEG]

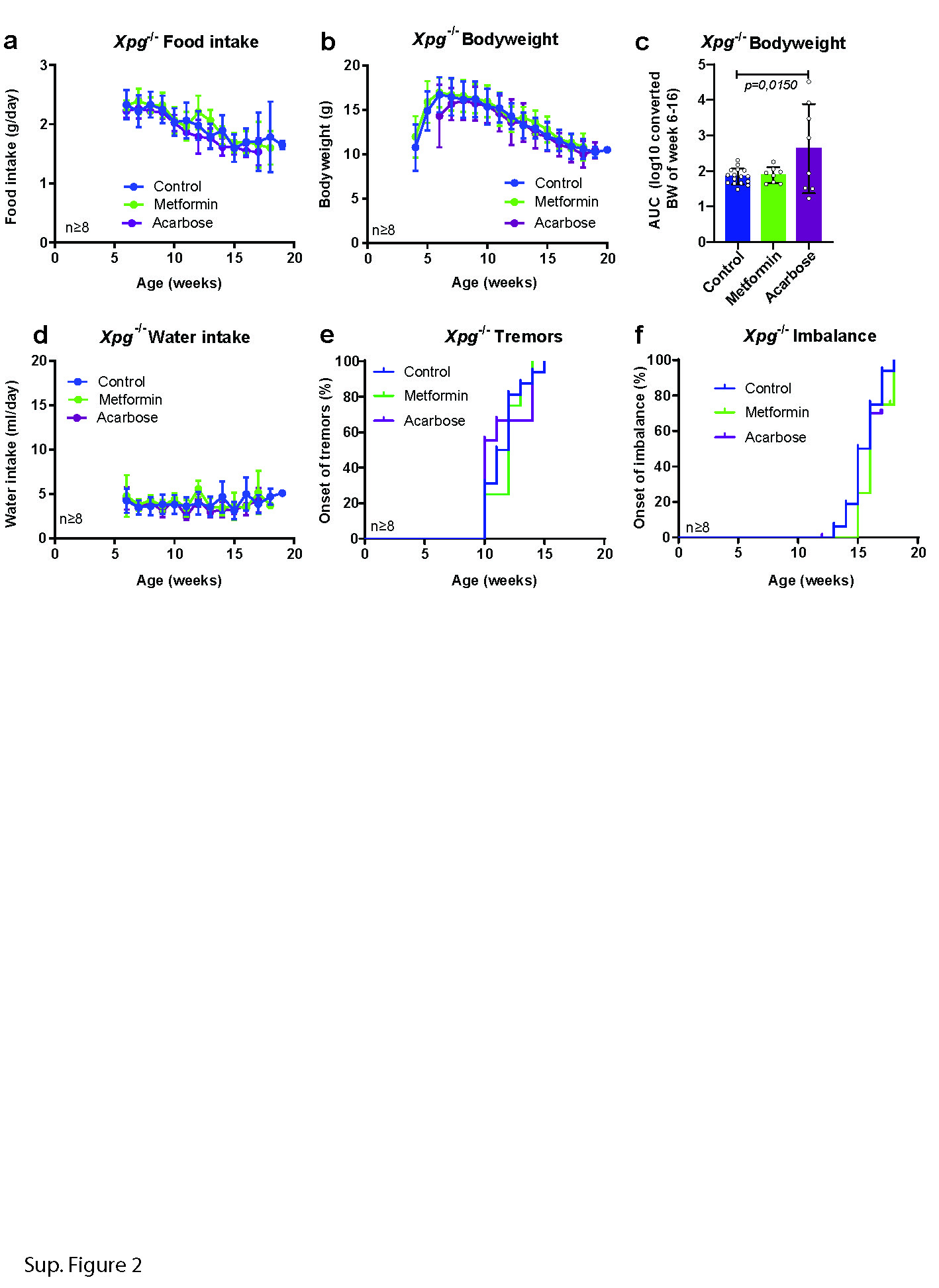

Supplement: Supplementary file 5 [file Image2.JPEG]

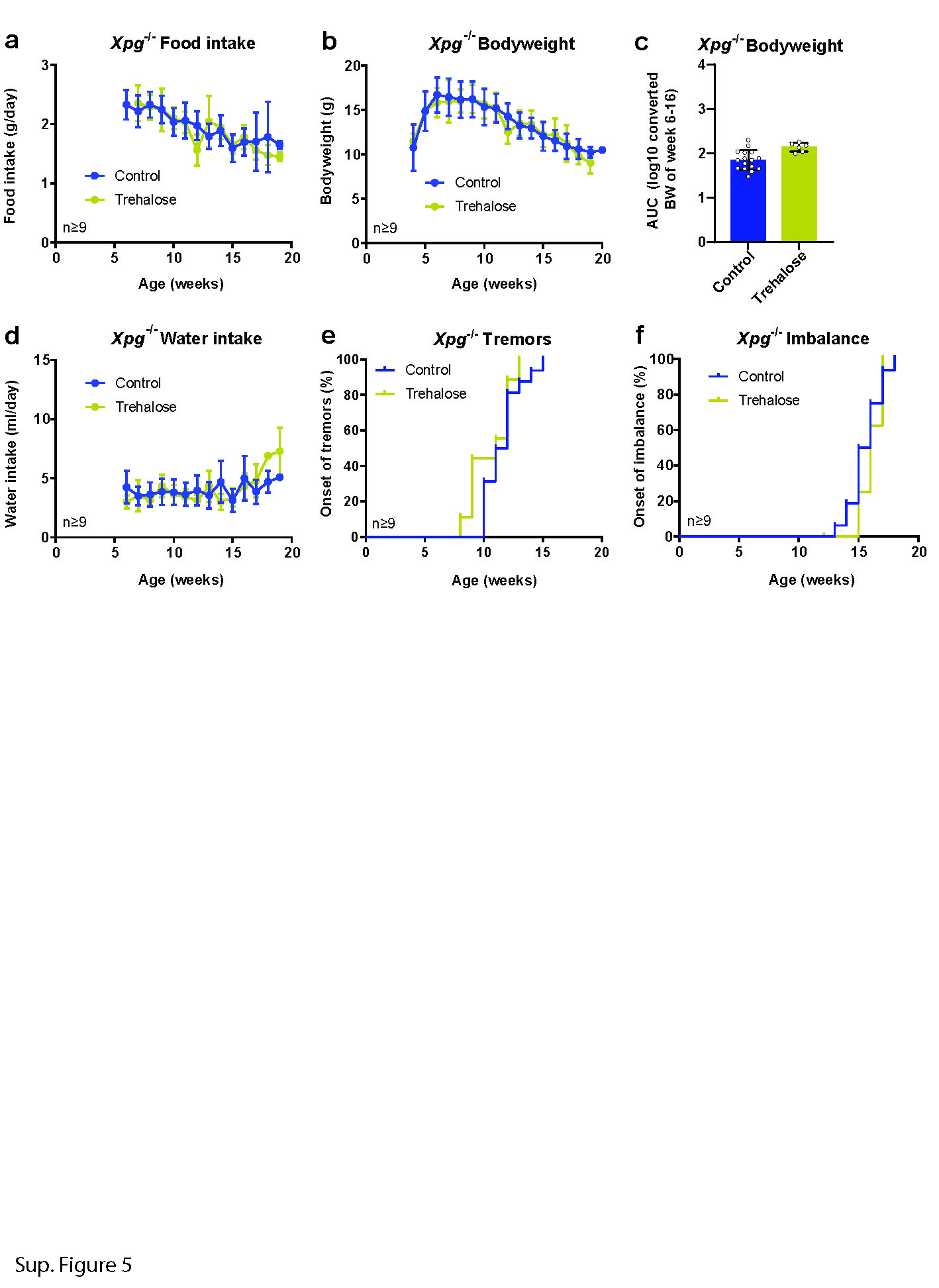

Supplement: Supplementary file 6 [file Image5.JPEG]

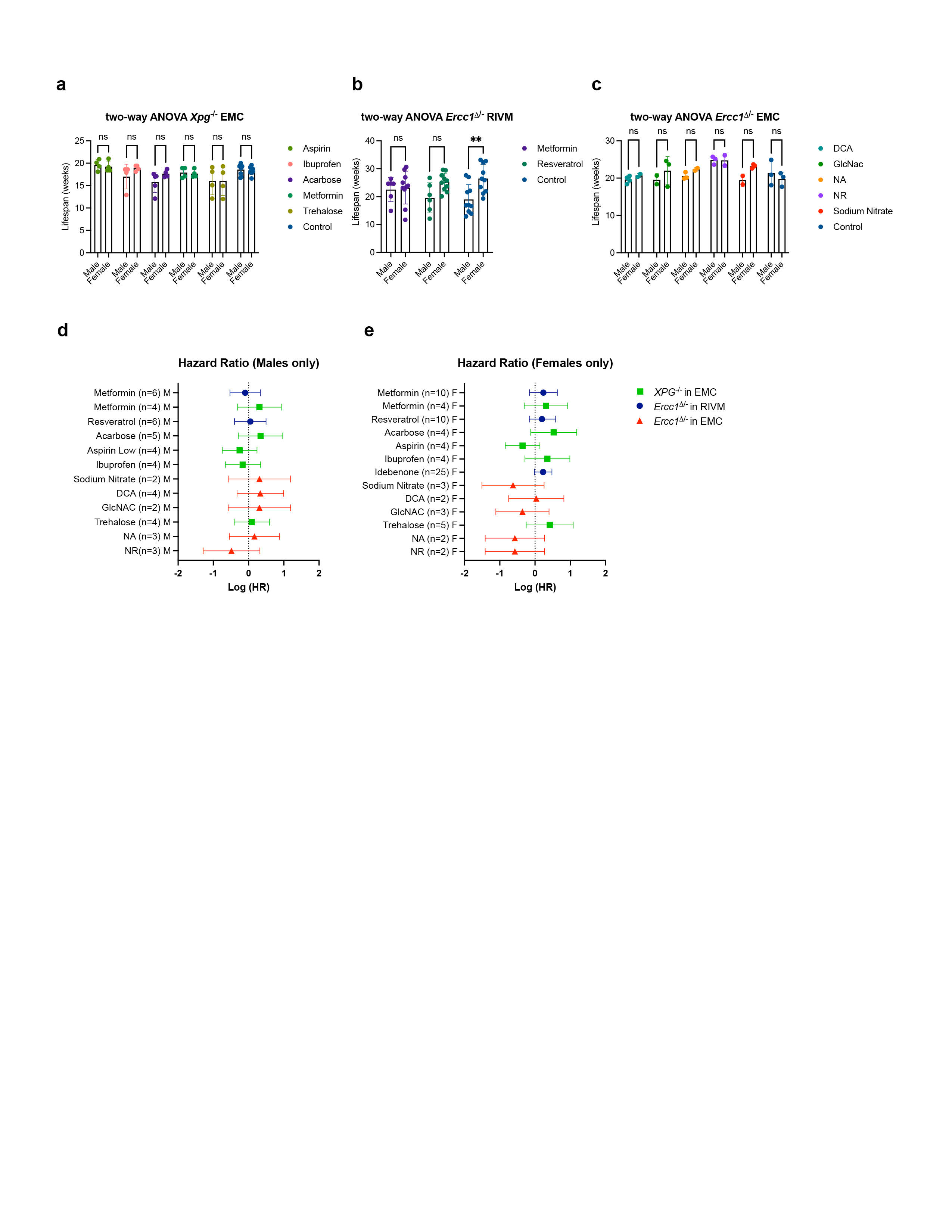

Supplement: Supplementary file 7 [file Image6.JPEG]
