# Supplementary material for: The use of progeroid DNA repair-deficient mice for assessing anti-aging compounds, illustrating the benefits of nicotinamide riboside
Source: Front Aging. 2022 Oct 12;3:1005322. doi: 10.3389/fragi.2022.1005322 (PMC9596940; doi:10.3389/fragi.2022.1005322)
Supplement: Supplementary file 2 [file Table1.docx]

|  | **Genotype** | **Location** | **Delivery** | **Start** | **Dose** | **Reference** | **N(M/F)** |
| --- | --- | --- | --- | --- | --- | --- | --- |
| **Nutrient sensing modulators** | |  |  |  |  |  |  |
| Metformin | *Ercc1*^Δ/-^ | RIVM | Food | 8 weeks | 1000 ppm | ^a^,(Strong et al., 2016) | 16 (6/10) |
|  | *Xpg*^-/-^ | EMC | Water | 4 weeks | 600 mg/l | ^a^,(Strong et al., 2016) | 8 (4/4) |
| Resveratrol | *Ercc1*^Δ/-^ | RIVM | Food | 8 weeks | 200 ppm | ^a^,(Strong et al., 2013) | 16 (6/10) |
| Acarbose | *Xpg*^-/-^ | EMC | Food | 4 weeks | 600 mg/l | ^a^,(Harrison et al., 2019) | 9 (5/4) |
| **Anti-inflammatory NSAID drugs** | |  |  |  |  |  |  |
| Aspirin | *Xpg*^-/-^ | EMC | Water | 4 weeks | 15 mg/l | ^a^,( Strong et al., 2008; Saleh et al., 2014) | 8 (4/4) |
| Ibuprofen | *Xpg*^-/-^ | EMC | Water | 4 weeks | 75 mg/l | (He et al., 2014) | 8 (4/4) |
| **Antioxidants** | | |  |  |  |  |  |
| Idebenone | *Ercc1*^Δ/-^ | RIVM | Food | 4 weeks | 743 ppm | ^b^ | 25 (0/25) |
| **Mitochondrial modulation** | | | | | | | |
| Sodium Nitrate | *Ercc1*^Δ/-^ | EMC | Water | 4 weeks | 50-100 mg/l* | ^b^,(Milanese et al., 2018) | 5 (2/3) |
| DCA | *Ercc1*^Δ/-^ | EMC | Water | 4 weeks | 1-2 mg/l* | ^b^ | 6 (4/2) |
| **Glucose homestasis modulators** | | |  |  |  |  |  |
| GlcNAc | *Ercc1*^Δ/-^ | EMC | Water | 4 weeks | 1-4 nM* | ^b^,(Denzel et al., 2014) | 5 (2/3) |
| Trehalose | *Xpg*^-/-^ | EMC | Water | 4 weeks | 2 mg/l | ^b^,(Khalifeh et al., 2021) | 9 (4/5) |
| **NAD precursors** |  |  |  |  |  |  |  |
| NR | *Ercc1*^Δ/-^ | EMC | Water | 4 weeks | 0,25-0,5 mg/l* | ^b^,(Scheibye-Knudsen et al., 2014 ) | 5 (3/2) |
| NA | *Ercc1*^Δ/-^ | EMC | Water | 4 weeks | 1-4 mg/l* | ^b^ | 5 (3/2) |

**Supplemental Table 1.** Overview of compounds, experimental location, delivery method, intervention start, concentration of drugs and number of male/female animals of each experimental group. Food concentrations of ppm were converted to concentrations in drinking water by using the average values for food intake of 2.1g/day and water intake of 5ml/day from both *Ercc1*^Δ/-^ and *Xpg*^-/-^ mice. *lowest dosage indicated was used from 4 weeks of age which was increased to the highest dose at 8 weeks of age for DCA and 10 weeks of age for sodium nitrate, GlcNAc, NR and NA. ^a^Dosage based on NIA ITP. ^b^Dosage based on own preliminary work.
